# Effects of high-intensity interval training on cardiorespiratory fitness in adolescents with overweight or obesity: a systematic review and dose–response meta-analysis

**DOI:** 10.3389/fphys.2026.1885059

**Published:** 2026-08-18

**Authors:** Jinchen Pan, Xusong Dong, Ruiqi Cheng, Jianmei Cui, Weian Lin, Lei Zheng

**Affiliations:** 1 China Basketball College, Beijing Sport University, Beijing, China; 2 Strength And Conditioning Training College, Beijing Sports University, Beijing, China; 3 School of Athletic Performance, Shanghai University of Sport, Shanghai, China; 4 College of Physical Education, North University of China, Taiyuan, China; 5 The Key Laboratory of Adolescent Health Assessment and Exercise Intervention of the Ministry of Education, East China Normal University, Shanghai, China

**Keywords:** adolescents, cardiorespiratory fitness, dose–response meta-analysis, high-intensity interval training, obesity, overweight

## Abstract

**Background:**

Adolescents with overweight or obesity commonly exhibit reduced cardiorespiratory fitness (CRF), which is closely associated with cardiometabolic risk and premature mortality in adulthood. High-intensity interval training (HIIT) has been proposed as a time-efficient exercise strategy; however, quantitative evidence regarding the dose of HIIT in adolescents with overweight or obesity remains limited. Objective: This study aimed to evaluate the effects of HIIT on CRF in adolescents with overweight or obesity and to explore the nonlinear association between weekly HIIT dose and improvements in CRF using dose–response meta-analysis.

**Methods:**

PubMed, Embase, Web of Science, Scopus, and CNKI were searched from database inception to March 2026. Eligible studies were randomized controlled trials involving adolescents aged 10–18 years with overweight or obesity. Interventions consisted of structured HIIT or sprint interval training, and comparators included usual care, health education, or no additional structured exercise. The outcomes were VO_2_max/VO_2_peak measured in the laboratory, or equivalent CRF indices estimated using standardized field tests. Two reviewers independently performed study screening, data extraction, and risk-of-bias assessment using the RoB 2 tool. Random-effects meta-analysis was used to Hedges’ g, and restricted cubic splines were further applied to examine the nonlinear relationship between weekly HIIT dose (MET-min/week) and CRF improvement.

**Results:**

Sixteen randomized controlled trials, including 19 effect sizes were included. Compared with control conditions, HIIT significantly improved CRF (Hedges’ g, 1.13, 95% CrI: 0.80–1.50). Exploratory subgroup analyses suggested more stable positive effects in studies using three sessions per week, a work-to-recovery ratio of approximately 1:1, and VO_2_max/VO_2_peak as the CRF outcome. Exploratory dose–response analysis suggested an inverted U-shaped association between weekly HIIT dose and CRF improvement, with the largest predicted effect observed at approximately 360 MET-min/week. However, this estimate should be interpreted cautiously because of the limited number of trials, between-study heterogeneity, and assumptions involved in MET-min/week conversion.

**Conclusions:**

HIIT may be an effective strategy for improving CRF in adolescents with overweight or obesity. Its benefits appear to depend not only on the use of HIIT per se, but also on training frequency, work–recovery structure, and weekly dose. Current evidence suggests a possible nonlinear association between weekly HIIT dose and CRF improvement; however, this finding is hypothesis-generating rather than definitive and should be verified in larger trials with standardized HIIT prescription reporting, transparent dose coding, and longer follow-up.

**Systematic review registrations:**

https://www.crd.york.ac.uk/PROSPERO, identifier CRD420261392042.

## 1 Introduction

Childhood and adolescent overweight and obesity have become among the most urgent public health challenges worldwide. According to the World Health Organization, in 2022, more than 390 million children and adolescents aged 5–19 years were living with overweight, including approximately 160 million with obesity (World Health Organization, 2024). Beyond excess body weight or adiposity alone, one of the most important manifestations of obesity-related health risk is the decline in physical fitness and cardiometabolic function (Lopez-Jimenez et al., 2022). Obesity during adolescence is not only associated with short-term mental health problems, sleep disturbance, musculoskeletal burden, and metabolic abnormalities, but may also increase the risk of type 2 diabetes, cardiovascular disease, and premature mortality in adulthood through the tracking of health behaviors, body composition, and cardiovascular risk factors (Reilly and Kelly, 2011; Hamilton et al., 2018).

Among obesity-related health indicators, cardiorespiratory fitness (CRF) has particular importance. CRF reflects the integrated capacity of the circulatory, respiratory, and skeletal-muscle systems to uptake, transport, and utilize oxygen during sustained physical activity, and is commonly expressed as maximal oxygen uptake (VO_2_max) or peak oxygen uptake (VO_2_peak) (Ross et al., 2016). Unlike body mass index, CRF is not only an indicator of physical performance but also an independent predictor of cardiometabolic health and all-cause mortality risk (Mintjens et al., 2018; Raghuveer et al., 2020; Kondamudi et al., 2021). The American Heart Association scientific statement identifies CRF as an important marker of current and future health in youth, with associations with cardiovascular health, mental health, cognitive function, and long-term health outcomes (Raghuveer et al., 2020). Therefore, improving CRF in adolescents with overweight or obesity has clinical and public health relevance that extends beyond weight control itself.

To improve CRF in adolescents with overweight or obesity, conventional public health guidelines often recommend moderate-intensity continuous training (MICT) (Liu et al., 2020; Huang et al., 2023). However, MICT typically requires a continuous time commitment of 45–60 min per session and may be perceived as monotonous. For adolescents who face substantial academic pressure, high levels of screen-based behavior, and low exercise self-efficacy, long-term adherence and sustainability may be limited (Zheng et al., 2025). In this context, high-intensity interval training (HIIT) has attracted considerable attention over the past decade as a highly time-efficient intervention (Costigan et al., 2015). By alternating short bouts of high-intensity work with low-intensity active recovery or passive rest, HIIT provides substantial cardiopulmonary and metabolic stimulation within a relatively short period, and is therefore considered a time-efficient strategy for improving CRF in adolescents (Eddolls et al., 2017; Martin-Smith et al., 2020). Some previous systematic reviews and meta-analyses suggest that HIIT may improve CRF in youth with overweight or obesity; however, direct comparisons between HIIT and MICT remain mixed, with some evidence indicating comparable or non-significant differences depending on intervention design, participant characteristics, comparator conditions, and outcome measurement (Eddolls et al., 2017; Martin-Smith et al., 2020; Deng and Wang, 2024). Therefore, the current evidence should not be interpreted as uniformly demonstrating the superiority of HIIT over MICT, but rather as supporting HIIT as a potentially effective and time-efficient alternative exercise strategy.

Although existing systematic reviews and meta-analyses have examined the effects of HIIT on CRF in adolescents, particularly those with overweight or obesity, several important gaps remain. First, many studies have focused primarily on the overall effect of HIIT relative to MICT or usual-care control conditions, while providing limited quantitative interpretation of key prescription components such as training frequency, work–recovery ratio, session duration, and intervention length. Second, substantial variation exists across HIIT protocols, suggesting that improvements in CRF may not increase linearly with training dose, but may instead involve threshold effects, plateau effects, or diminishing marginal returns at higher doses (Deng and Wang, 2024). Third, adolescents with overweight or obesity are heterogeneous in baseline CRF, degree of obesity, pubertal development, and exercise tolerance, which may lead to differential responses to HIIT dose.

Previous reviews have established that HIIT can improve CRF in adolescents and have examined potential moderators of its effects, including intervention duration, training modality, intensity, frequency, and accumulated HIIT volume (Costigan et al., 2015; Eddolls et al., 2017; Martin-Smith et al., 2020). However, these reviews primarily estimated the overall intervention effect or examined individual protocol characteristics separately, rather than modelling an integrated weekly HIIT dose as a continuous nonlinear exposure (Martin-Smith et al., 2020; Deng and Wang, 2024). However, their dose-related conclusions were derived mainly from categorical subgroup analyses and conventional meta-regression of intervention duration, number of sets, work-bout duration, recovery duration, and work–recovery ratio. Similarly, a previous review in adults with overweight or obesity examined dose–response relationships for separate HIIT prescription variables but concluded that sufficiently precise dose recommendations could not yet be established (Wang et al., 2021).

The present review therefore extends, rather than duplicates, previous work by standardizing the intensity and duration of different HIIT protocols as weekly MET-min/week and applying Bayesian multilevel spline models to examine the shape of the association between weekly HIIT dose and CRF improvement in adolescents with overweight or obesity. Given the substantial variation in HIIT protocols and evidence that increasing individual training components does not necessarily produce progressively greater benefits (Wang et al., 2021; Deng and Wang, 2024), the association may not follow a simple linear pattern. Instead, threshold effects, plateau effects, or diminishing marginal returns at higher weekly doses may be present.

Against this background, this systematic review and dose–response meta-analysis aimed to evaluate the effects of HIIT on CRF in adolescents with overweight or obesity and to explore the dose–response relationship between weekly HIIT dose and the magnitude of CRF improvement. By focusing on both overall effectiveness and prescription-related dose patterns, this review may provide more practice-oriented evidence for designing feasible, time-efficient, and appropriately dosed HIIT interventions for adolescents with overweight or obesity.

## 2 Methods

### 2.1 Registration

This systematic review and meta-analysis was designed, conducted, and reported in accordance with the Preferred Reporting Items for Systematic Reviews and Meta-Analyses (PRISMA 2020) statement (Page et al., 2021). The study protocol was prospectively registered in the International Prospective Register of Systematic Reviews (PROSPERO; registration number: CRD420261392042, https://www.crd.york.ac.uk/PROSPERO/view/CRD420261392042).The PRISMA checklist was presented in **Supplementary Table S1**.

### 2.2 Literature search strategy

Two researchers independently searched five electronic databases: PubMed, Embase, Web of Science Core Collection, Scopus, and China National Knowledge Infrastructure (CNKI). Searches covered all records from database inception to March 2026. The search strategy combined controlled vocabulary terms and free-text terms. Search terms were constructed around four core concepts: (1) intervention: “High-Intensity Interval Training” OR “HIIT” OR “High-Intensity Intermittent” OR “Sprint Interval Training” OR “SIT” OR “Aerobic Interval Training” OR “Interval exercise”; (2) outcomes: “Cardiorespiratory fitness” OR “CRF” OR “Maximal oxygen uptake” OR “Peak oxygen uptake” OR “VO2max” OR “VO2peak” OR “Aerobic capacity” OR “Cardiovascular fitness”; (3) population: “Adolescent” OR “Youth” OR “Teenager” OR “Puberty” OR “High school student”; and (4) overweight or obesity status: “Obesity” OR “Overweight” OR “Obese” OR “Adiposity” OR “Body weight”.

The search strings were adapted to the subject headings and field rules of each database and are reported in full in the supplementary materials. To reduce the risk of missing eligible studies, the reference lists of included studies and relevant high-quality reviews were also manually screened. All retrieved records were imported into EndNote reference management software, and duplicates were removed using automated procedures supplemented by manual verification. The detailed search strategy for each database was presented in **Supplementary Table S2**.

### 2.3 Eligibility criteria

Eligibility criteria were defined according to the PICOS framework. Population: adolescents aged 10–19 years with overweight or obesity, diagnosed according to WHO, CDC, IOTF, or nationally/regionally accepted age- and sex-specific BMI criteria. Intervention: structured HIIT or sprint interval training (SIT), consisting of repeated alternation between high-intensity work bouts and low-intensity active recovery or passive rest, with clearly reported intensity-monitoring methods. Comparator: no additional structured exercise, usual care, health education, or regular school physical education. Outcomes: quantitative CRF-related outcomes, including laboratory-measured VO_2_max/VO_2_peak or CRF indices estimated from validated field tests. Study design: randomized controlled trials, including parallel-group RCTs; for crossover trials, first-period data were preferentially extracted or appropriate methods were used to account for the correlation inherent in crossover designs.

Exclusion criteria were as follows: observational studies, nonrandomized pre–post controlled studies, single-arm studies, conference abstracts, reviews, and case reports; studies involving professional competitive athletes, adolescents with secondary obesity, or adolescents with severe cardiopulmonary disease; studies in which HIIT was embedded in a complex multicomponent intervention and its independent effect could not be isolated; and studies in which the control group received structured training, such as resistance training, that was not aligned with the research question and did not permit estimation of the effect of HIIT versus usual-care control conditions. No additional search of trial registries, or grey literature sources was conducted. Studies were limited to full-text peer-reviewed publications in English or Chinese. Conference abstracts, dissertations, protocols without outcome data, and unpublished reports were excluded.

### 2.4 Study selection

Study selection was conducted in two stages. First, two independent reviewers screened titles and abstracts. Second, the full texts of potentially eligible records were retrieved and assessed in detail. Any disagreements between reviewers were resolved through discussion; if disagreement persisted, a senior third reviewer was consulted to make the final decision.

### 2.5 Data extraction

Data extraction was performed independently by two investigators using a predesigned standardized Excel extraction form. Any ambiguity during extraction was adjudicated by a third researcher. If key raw data were missing or unclear in the published articles, the study team contacted the corresponding authors by email at least twice to obtain unpublished supplementary data. Extracted information included demographic data (sample size, mean age, sex distribution, baseline BMI, and pubertal stage), intervention characteristics (total duration, weekly frequency, session duration, and work–recovery ratio), and outcome parameters, including the sample size, mean, and standard deviation (SD) for CRF and secondary outcomes at baseline and post-intervention in the intervention and control groups.

### 2.6 Dose coding

To compare different HIIT protocols, weekly training dose was standardized as MET-min/week. The basic formula was: weekly HIIT dose, Σ(work-bout MET × work-bout duration in minutes + recovery-bout MET × recovery-bout duration in minutes) × weekly training frequency. When a session included multiple repeated intervals, the duration of all work bouts and planned recovery bouts was summed separately before multiplying by the assigned MET values. When the intervention dose progressed during the study period, dose was calculated for each reported phase and averaged across the intervention period. Warm-up and cool-down periods were not included in the primary HIIT dose calculation unless they were explicitly described as part of the interval-training prescription, because these components were not consistently reported and were not specific to the HIIT stimulus. MET values were assigned primarily according to the 2011 Compendium of Physical Activities and the exercise-intensity information reported in each study (Ainsworth et al., 2011). MET values were assigned using a prespecified hierarchical coding procedure applied separately to work bouts and recovery bouts. When the original trial directly reported MET values or energy-expenditure estimates, these values were used preferentially. When MET values were not reported, intensity information based on heart rate, percentage of maximal oxygen uptake, speed, power, or rating of perceived exertion (RPE) was used to classify the bout into the closest corresponding intensity category and then matched to the most appropriate Compendium activity code. If several intensity indicators were reported, physiological indicators were prioritized over subjective indicators. If only broad descriptors such as “high intensity,” “all-out,” or “active recovery” were available, conservative MET assignments were made according to the reported exercise modality and intensity description. All dose-coding decisions were recorded at the intervention-arm level, including exercise modality, work-bout intensity, recovery-bout intensity, monitoring method, assigned work-bout MET, assigned recovery-bout MET, weekly frequency, calculated MET-min/week, and coding assumptions. The hierarchical coding rules are summarized in **Supplementary Table S3**.

### 2.7 Risk-of-bias assessment

Two independent reviewers assessed risk of bias using the revised Cochrane risk-of-bias tool for randomized trials (RoB 2.0) (Sterne et al., 2019). RoB 2.0 evaluates five core domains: bias arising from the randomization process; bias due to deviations from intended interventions; bias due to missing outcome data; bias in measurement of the outcome; and bias in selection of the reported result. Based on responses to a series of signaling questions, studies were rated as having “low risk of bias”, “some concerns”, or “high risk of bias”. An overall risk-of-bias judgment was then derived for the primary outcome of each study. Any disagreements were resolved through discussion.

### 2.8 Statistical analysis

All statistical analyses were conducted in R software (version 4.3.0), primarily using the metafor, meta, brms, and ggplot2 packages. All tests were two-sided, with P < 0.05 considered statistically significant.

#### 2.8.1 Main meta-analysis

The standardized mean difference (SMD) and its 95% credible interval (CrI) were used as the pooled effect measure, and Hedges’ g was applied to correct for small-sample bias (Hedges, 1981). Effect sizes were calculated using post-intervention CRF values where available; when post-intervention values were not available, change-score data were used. Given clinical and methodological heterogeneity across studies in participant characteristics, HIIT protocols, and CRF measurement methods, a Bayesian random-effects model was used for quantitative synthesis. The Bayesian random-effects model combines prior information with Markov chain Monte Carlo (MCMC) simulation to estimate the probability distribution of effect sizes and to account for between-study heterogeneity and uncertainty. The model was fitted in R using the brms package (Bürkner, 2017).

For the three-arm trial that included both HIIT and SIT interventions sharing a common control group, separate HIIT–control and SIT–control effect sizes were calculated because the intervention arms represented distinct high-intensity interval prescriptions. The data were retained in an arm-based format, and the control-group sample size was not split in the primary analysis. Both effect sizes were assigned the same Study_ID and separate effect-size identifiers and were analysed using a multilevel model specified as (1 | Study_ID/esid), thereby clustering effect sizes within study rather than treating them as estimates from independent trials.

Weakly informative priors were specified, with the overall intercept assigned a normal distribution N(0,1) and the between-study heterogeneity parameter τ assigned a half-Cauchy distribution Half-Cauchy (0,1) (Lemoine, 2019). The N(0,1) prior was chosen because very large standardized effects are uncommon in exercise-intervention meta-analyses, while this prior still allows moderate-to-large positive or negative effects. The Half-Cauchy (0,1) prior was used because τ is constrained to positive values and substantial heterogeneity was plausible given differences in participants, interventions, and outcome measures. Prior-sensitivity analyses using alternative weakly informative priors were conducted to examine whether the main conclusions were sensitive to prior specification. Each model was estimated using four Markov chains with 10,000 iterations per chain, and convergence was assessed using Rhat values and trace plots. Posterior sampling quality was additionally evaluated using bulk effective sample size (bulk-ESS) and tail effective sample size (tail-ESS), particularly for the pooled effect and the heterogeneity parameter τ. Heterogeneity was quantified by examining the posterior distribution of the random-effects standard deviation (τ) and I². The results for Bayesian model diagnostics are presented in **Supplementary Table S4**.

#### 2.8.2 Dose–response meta-analysis

To investigate the association between HIIT dose and CRF improvement, weekly cumulative exercise dose (MET-min/week) was used as the exposure variable and Hedges’ g as the outcome variable. Linear and nonlinear dose–response models were constructed. Nonlinear associations between weekly HIIT dose and CRF improvement were examined using spline-based Bayesian dose–response models. Given the limited number of included trials and the risk of overfitting, the primary nonlinear model used a penalized spline smooth term for weekly HIIT dose with a low basis dimension (k, 3). To assess whether the observed dose–response pattern depended on the selected functional form, additional candidate models were fitted, including a k, 4 penalized spline smooth model, restricted cubic spline models with three and four knots, quadratic and cubic polynomial models, a log-dose model, natural spline models, and B-spline models. Candidate models were compared using approximate leave-one-out cross-validation. Model selection considered both predictive performance and parsimony.

#### 2.8.3 Meta-regression and subgroup analyses

To explore potential sources of heterogeneity, several prespecified moderators were examined using Bayesian meta-regression, including degree of obesity, baseline CRF level, proportion of female participants, HIIT type, HIIT modality, and work-to-recovery time ratio. Effect estimates were expressed as SMDs with 95% CrIs.

#### 2.8.4 Sensitivity analyses and publication bias

Sensitivity analyses were conducted by sequentially excluding one study at a time to assess the robustness of the pooled effect. Additional sensitivity analyses included prior-sensitivity analysis, and the four-knot spline model for the dose–response analysis. Prior-sensitivity analyses were conducted to examine whether the main findings were sensitive to prior specification. The primary model used a Normal(0,1) prior for the pooled effect and a Half-Cauchy(0,0.5) prior for the heterogeneity parameters. Alternative models used a wider Normal(0,2) prior for the pooled effect, a wider Half-Cauchy(0,1) prior for heterogeneity, and a Half-Normal(0,0.5) prior for heterogeneity. Model convergence and posterior sampling quality were assessed using Rhat, bulk effective sample size (bulk-ESS), and tail effective sample size (tail-ESS) for the pooled effect and heterogeneity parameters.

Publication bias was evaluated using funnel plots, Egger’s regression test, and Begg’s rank correlation test (Egger et al., 1997). If potential publication bias was detected, the trim-and-fill method was further applied to adjust the pooled estimate (Duval and Tweedie, 2000). Because conventional funnel-plot methods, Egger’s regression test, Begg’s rank correlation test, and trim-and-fill analysis do not directly accommodate multilevel meta-analytic structures with statistically dependent effect sizes, publication-bias analyses were conducted using study-level aggregated effect sizes. For studies contributing more than one eligible HIIT comparison, effect sizes were aggregated within study before conducting publication-bias analyses. Publication-bias analyses were interpreted as sensitivity analyses rather than definitive evidence of bias. Where trim-and-fill adjustment was applied, both the unadjusted and adjusted pooled estimates were reported to show the potential degree of change in the overall effect.

## 3 Results

### 3.1 Study selection

**Figure 1:**
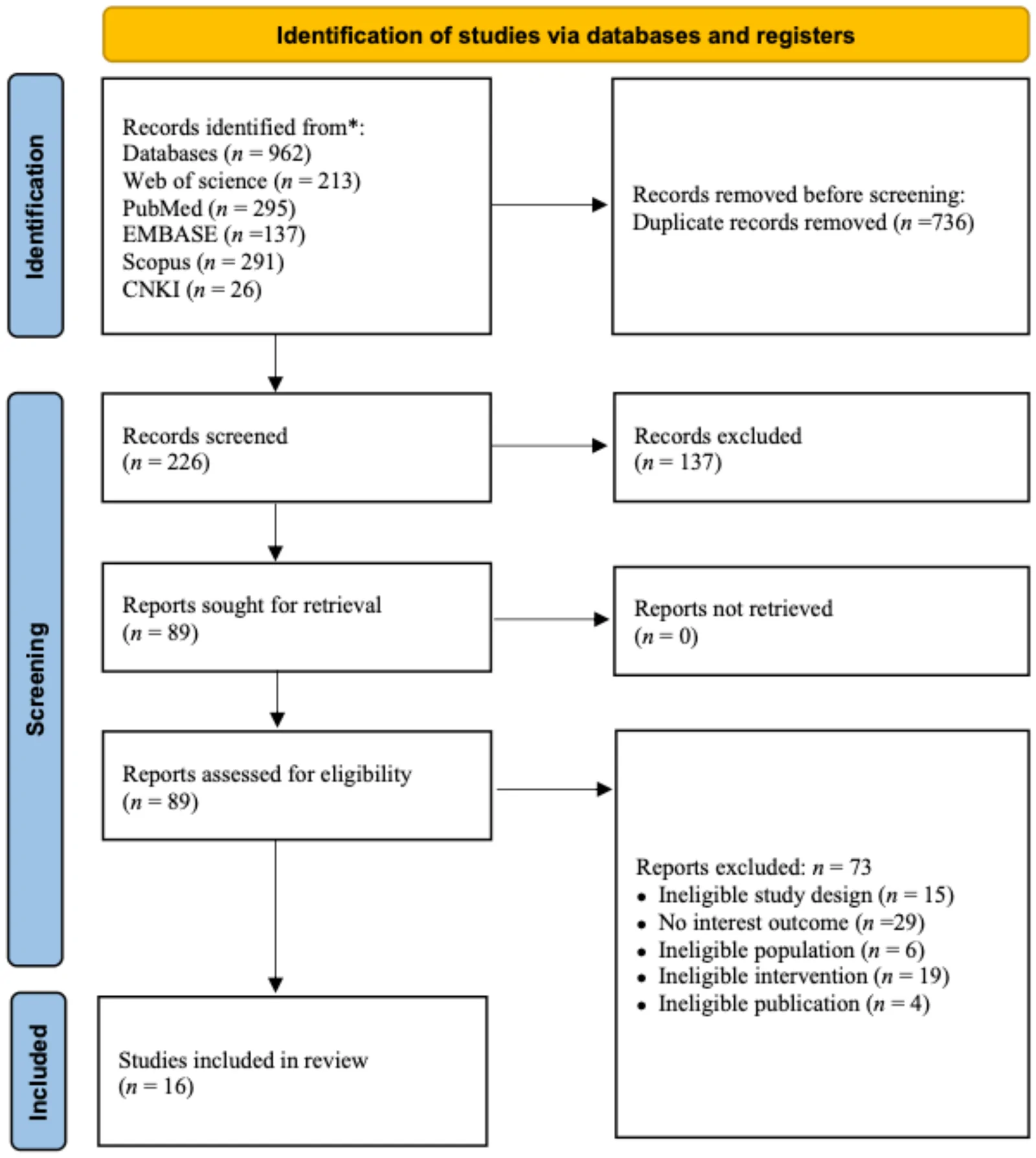
PRISMA flow diagram.

The database searches yielded 962 records. After removal of duplicates, 226 records remained. Following title and abstract screening, 89 articles underwent full-text assessment. Based on the predefined inclusion and exclusion criteria, 16 randomized controlled trials were ultimately included in the quantitative synthesis. The study selection process is presented in **Figure 1**.

### 3.2 Characteristics of included studies

The 16 included RCTs were published between 2013 and 2025 and contributed 19 effect estimates. The number of effect estimates exceeded the number of studies because two studies each reported CRF using both relative VO_2_peak (mL/kg/min) and absolute VO_2_peak (L/min), while one three-arm study contributed separate HIIT–control and SIT–control comparisons. Individual study sample sizes ranged from 20 to 92 participants, and the mean participant age ranged mainly from 10.6 to 18.0 years. The interventions lasted 6–12 weeks, with 12-week protocols being the most common, and most studies delivered three training sessions per week. The HIIT protocols varied in exercise modality, intensity, work-bout duration, and recovery structure, with a 1:1 work–recovery ratio being the most frequently used. CRF was assessed predominantly using VO_2_max or VO_2_peak expressed in mL/kg/min; two studies additionally reported absolute VO_2_peak in L/min, and three studies used a Yo-Yo field test. Detailed characteristics of the included studies are presented in **Table 1**.

**Table 1:** Characteristics of included randomized controlled trials.

Study	Country	Design	Participants	Female(%)	BMI	HIIT protocol	Comparator	CRF outcome
Khammassi 2018 (Khammassi et al., 2018)	Tunisia	RCT	n, 20; overweight/obese sedentary males; age 18 years	0	29.3	12 weeks; 3 sessions/week; 30 s at 100% MAV interspersed with 30 s active recovery at 50% MAV; repetitions progressed from 15 to 27	Non-exercise control	VO_2_max (ml/kg/min)
Lau 2015 (Lau et al., 2015)	China	RCT	n, 27; overweight children; age 11.0/10.6 years	NR	23.7/24.8	6 weeks; 3 sessions/week; HIIT: 12 × 15 s at 120% MAS with 15 s passive recovery	No scheduled training control	Yo-Yo
Leite 2022 (Leite et al., 2022)	Brazil	RCT	n, 36 obese boys; age 10–16 years; HIIT n, 20, CG n, 16	0	30.67/30.71	12 weeks; 3 sessions/week; HIIT sessions included 15 min warm-up, 15–18 min interval exercise, and 15 min cool-down	Non-exercise control	VO_2_peak (ml/kg/min)
Meng 2022 (Meng et al., 2022)	China	RCT	n, 30 obese boys; age 11.2 ± 0.7 years; HIIT n, 15, CG n, 15	0	24.5/23.8	12 weeks; 3 sessions/week; 2 sets of 8 × 15 s running at 90–100% MAS with 15 s recovery running at 50% MAS	Non-exercise control	VO_2_peak (ml/kg/min)
Racil 2013 (Racil et al., 2013)	Tunisia	RCT	n, 23 obese adolescent girls; age 15.9 ± 0.3 years; HIIT n, 11, CG n, 12	100	30.8/30.8	12 weeks; high-intensity interval running; 30 s work bouts at high intensity with active recovery; protocol based on MAS/VO_2_peak-related speed	Non-exercise control	VO_2_peak (ml/kg/min)
Racil 2016 (Racil et al., 2016)	Tunisia	RCT	n, 42 obese female adolescents; age 16.6 ± 1.3 years; HIIT n, 23, CG n, 19	100	30.8/30.8	12 weeks; HIIT: 2 blocks/session of 6–8 × 30 s runs at 100% vVO_2_peak with 30 s active recovery at 50% vVO_2_peak	Non-exercise control	VO_2_peak (ml/kg/min)
Tadiotto 2023 (Tadiotto et al., 2023)	Brazil	RCT	n, 37 overweight adolescents; both sexes; age 11–16 years; HIIT n, 13, CG n, 24	NR	NR	12 weeks; 3 weekdays/week; HIIT on stationary bicycle; session lasted ~35 min	Non-exercise control	VO_2_peak (ml/kg/min)/VO_2_peak (L/min)
Li 2023 (Li et al., 2023)	China	RCT	n, 60 obese children; age 11.0 ± 0.8 years; 30 boys and 30 girls	50	24.2/23.6	12 weeks; HIIT at 100–110% MAS	Non-exercise control	VO_2_max (ml/kg/min)
Bogataj 2021 (Bogataj et al., 2021)	Serbia	RCT	n, 48 overweight adolescent girls; HIIT n, 24, CG n, 24	100	NR	8 weeks; 3 sessions/week; 10 stations of bodyweight HIIT; combined with nutrition education twice/week	Non-exercise control	Yo-Yo
Cao 2023 (Meng et al., 2022)	China	RCT	n, 40 obesity adolescents; age 11.0 ± 0.6 years; 20 boys; HIIT n, 20, CG n, 20	50	23.4/23.8	12 weeks; 3 sessions/week; 18 min/session; 3 sets of 8 × 15 s running at 100% MAS with 15 s recovery at 50% MAS	Non-exercise control	VO_2_max (ml/kg/min)
Chuensiri 2018 (Chuensiri et al., 2018)	Thailand	RCT	n, 22 obese adolescent boys aged 11 years; final analysis: CG n, 11, HIIT n, 11	0	24.2/26.1	12 weeks; 3 sessions/week. HIIT: 8 × 2 min cycling at 90% peak power output with 1 min passive recovery.	Sedentary usual-activity control	VO_2_peak (ml/kg/min)
Cvetković 2018 (Cvetković et al., 2018)	Serbia	RCT	n, 28 overweight/obese boys; age 11–13 years; HIIT n, 14, CG n, 14	0	25.4/25.5	12 weeks; progressive HIIT running protocol; work–rest duration progressed from 10:10 s to 15:15 s and 20:20 s; 100% MAS during work bouts	Non-exercise control	Yo-Yo
Domaradzki 2025 (Domaradzki et al., 2025)	Poland	RCT	n, 92 overweight adolescents; age 15.1 years; HIIT n, 45, CG n, 47	0	21.2/21.0	8 weeks; 2 sessions/week; progressively increased from 4 to 8 rounds of 20 s intense effort/10 s rest; HIIT based on bodyweight resistance exercises	Non-exercise control	VO_2_max (ml/kg/min)
González-Gálvez 2024 (González-Gálvez et al., 2024)	Spain	RCT	n, 45 recruited; final n, 32 overweight/obese adolescents; SIT n, 9, HIIT n, 11, CG n, 12; age 12.51 ± 0.75 years	NR	22.9	8 weeks; 2 sessions/week; 12 min/session; HIIT: 3 × 2 min at 80–85% HRmax with 2 min recovery at 50–55% HRmax; SIT: 6 × 60 s at 90–95% HRmax with 60 s recovery at 50–55% HRmax	Non-exercise control	VO_2_max (ml/kg/min)
Dias 2018 (Dias et al., 2018)	Australia	RCT	n, 67 obesity; age 12 years; HIIT n, 33, CG n, 34	NR	29.5/29.5	12 weeks; 3 sessions/week; 4 × 4 min at 85–95% HRmax with 3 min active recovery at 50–70% HRmax	Non-exercise control	VO_2_peak (ml/kg/min)/VO_2_peak (L/min)
Cao 2022 (Cao et al., 2022)	China	RCT	n, 40 children with obesity; age 11.2 years; 20 boys and 20 girls; HIIT n, 20, CG n, 20	50	24.3/23.8	12 weeks; 3 sessions/week; HIIT at 100–120% MAS	Non-exercise control	VO_2_max (ml/kg/min)

IG, intervention group; CG, control group; NR, not reported. Values after the slash for age and BMI refer to the control group when different from the intervention group. Tadiotto 2023, González-Gálvez 2024, and Dias 2018 each included two HIIT arms (a/b) sharing a common control group; sample sizes are listed as reported.

### 3.3 Risk-of-bias assessment

**Figure 2:**
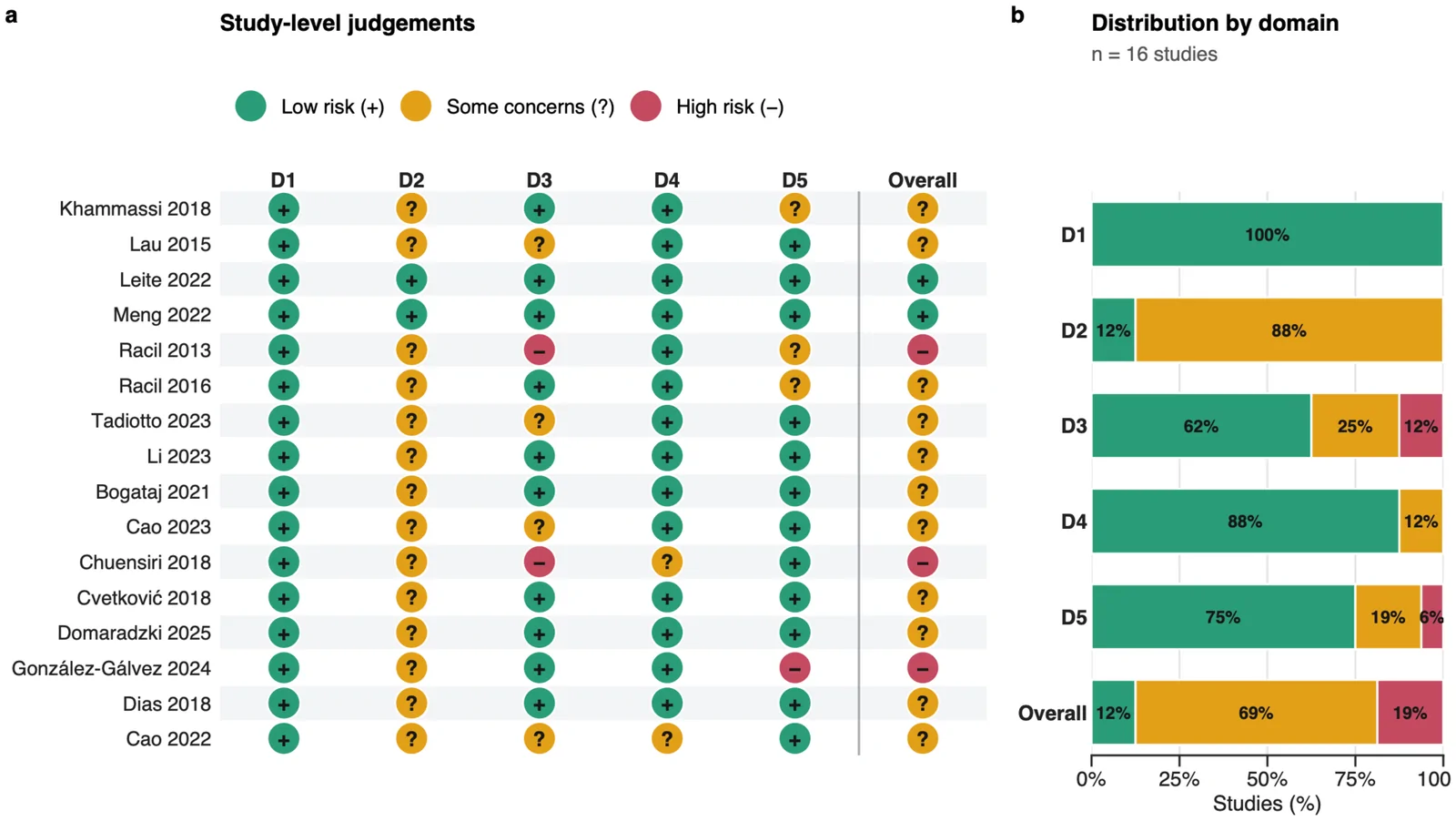
Risk-of-bias assessment.

Risk of bias in the included studies was assessed using the Cochrane RoB 2.0 tool. Overall, the methodological quality of the included studies was moderate (**Figure 2**). The overall risk-of-bias judgments were predominantly “some concerns”, with some studies rated as high risk and only a few rated as low risk. Across individual domains, D1 (randomization process) and D2 (deviations from intended interventions) generally performed well and were mostly judged as low risk, although a small number of studies raised some concerns for D2. D4 (measurement of the outcome) showed the best performance, with almost all studies judged as low risk, indicating that the measurement of CRF outcomes was generally objective and reliable. In contrast, D3 (missing outcome data) and D5 (selection of the reported result) were the main sources of risk of bias; both were dominated by “some concerns”, with a small number of high-risk judgments.

### 3.4 Main effect

**Figure 3:**
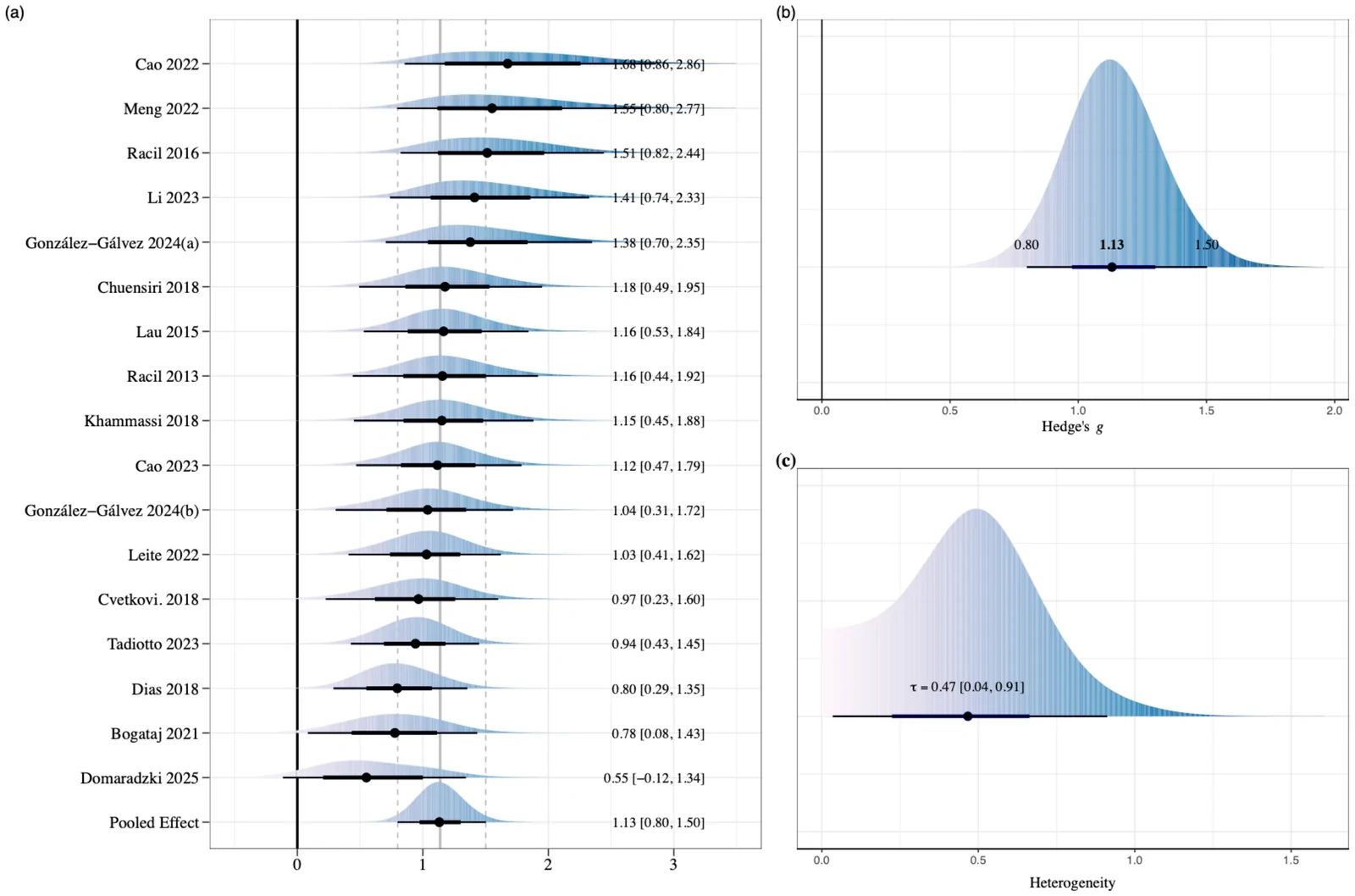
Main effect.

The random-effects model showed that HIIT significantly improved CRF in adolescents with overweight or obesity compared with control conditions (**Figure 3**). The pooled effect size was Hedges’ g, 1.13 (95% CrI: 0.80 to 1.50). The posterior probability that HIIT was superior to control exceeded 99.9%. The heterogeneity parameter τ was 0.47 (95% CrI: 0.04 to 0.91), and I² was approximately 62.5%, indicating moderate between-study heterogeneity.

### 3.5 Dose–response relationship

**Figure 4:**
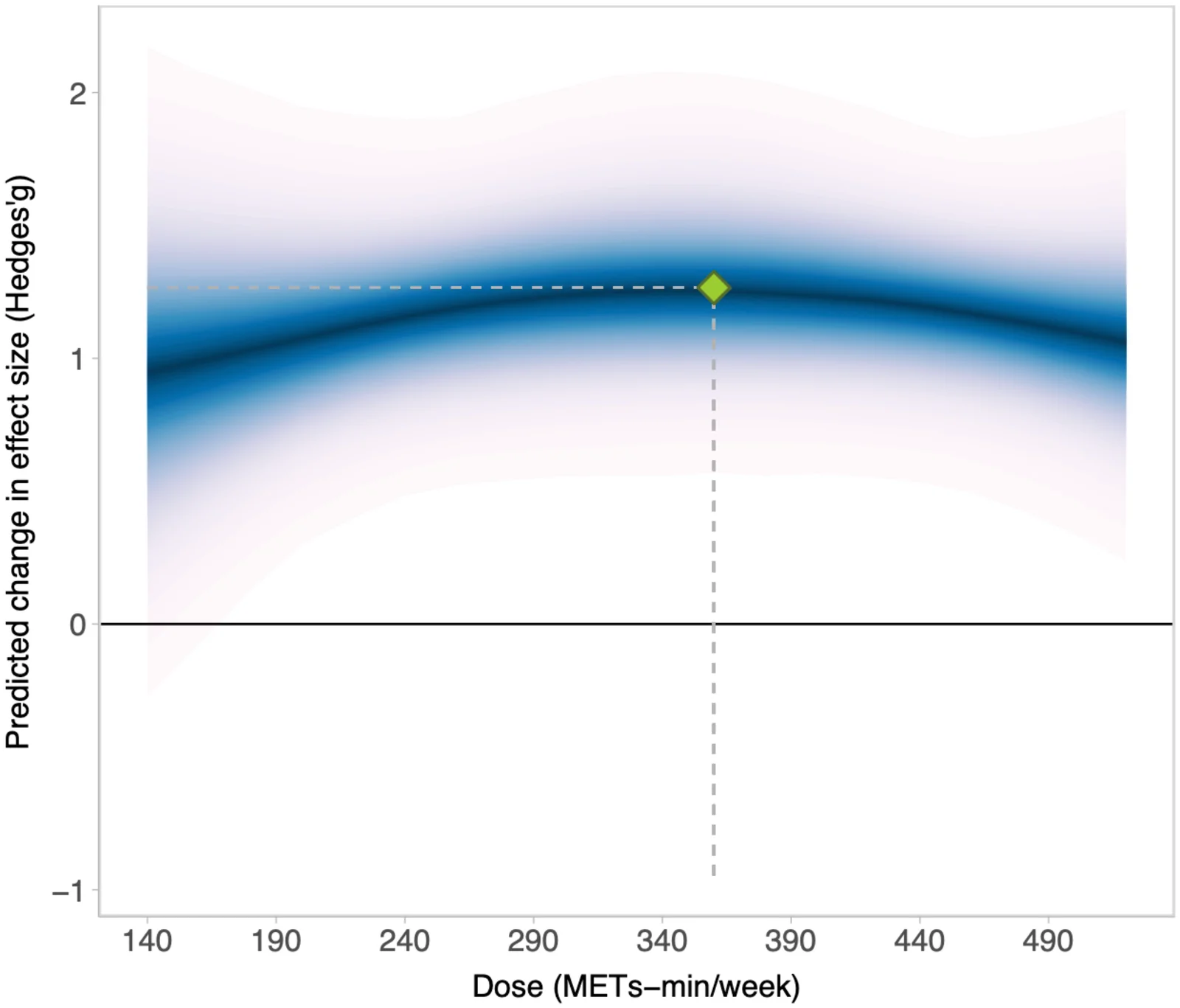
Dose–response relationship.

Approximate leave-one-out cross-validation was used to compare alternative nonlinear dose–response models (**Supplementary Table S5**). The four-knot restricted cubic spline model had the highest expected log predictive density and the lowest LOOIC among the candidate models. However, its advantage over the k, 3 penalized spline model was negligible relative to the standard error of the difference (elpd_diff, -0.2, SE, 0.8). Similar negligible differences were also observed for the k, 4 penalized spline model and the three-knot restricted cubic spline model. Therefore, given the limited number of trials and the need to avoid overfitting, the more parsimonious k, 3 penalized spline model was retained as the primary nonlinear dose–response model. The model indicated a nonlinear association between weekly HIIT dose and CRF improvement (**Figure 4**). As weekly dose increased from a low to a moderate level, the predicted effect size gradually increased. When weekly dose exceeded a certain level, the effect size did not increase further but instead plateaued or showed a slight decline. The predicted curve suggested that the peak effect occurred at approximately 360 MET-min/week, corresponding to a predicted effect size of approximately Hedges’ g, 1.25. This finding suggests that the CRF benefits of HIIT may involve an optimal dose window rather than a simple “more is better” pattern. The dose–response analysis was interpreted as exploratory and hypothesis-generating rather than as confirmatory evidence of an optimal dose threshold.

### 3.6 Subgroup analyses

Exploratory subgroup analyses were conducted to examine whether the pooled effect differed by training frequency, work–recovery ratio, and CRF measurement method (**Tables 2**, **3**). Because the number of trials and effect sizes within some subgroup categories was limited, these findings should be interpreted as hypothesis-generating rather than confirmatory.

**Table 2:** Exploratory subgroup analysis.

Subgroup variable	Category	No. of studies	No. of effect sizes	Hedges’ g	95% CrI
Training frequency	2 sessions/week	2	3	0.80	−0.03 to 1.67
	3 sessions/week	14	16	1.20	0.83 to 1.62
Work–recovery ratio	1:1	9	11	1.34	0.87 to 1.85
	1:2	2	3	0.81	−0.16 to 1.76
	2:1	5	5	0.91	0.26 to 1.61
CRF measurement	VO_2_max	6	7	1.29	0.70 to 1.90
	VO_2_peak	7	9	1.18	0.64 to 1.76
	Yo-Yo test	3	3	0.73	−0.13 to 1.58

**Table 3:** subgroup contrasts.

Subgroup variable	Subgroup contrast	Difference in Hedges’ g	95% CrI	Interpretation
	VO_2_peak vs VO_2_max	−0.11	−0.95 to 0.72	No clear subgroup difference
CRF measurement	Yo-Yo test vs VO_2_max	−0.56	−1.63 to 0.47	No clear subgroup difference
	Yo-Yo test vs VO_2_peak	−0.45	−1.48 to 0.56	No clear subgroup difference
Work–recovery ratio	1:1 vs 1:2	0.54	−0.53 to 1.64	No clear subgroup difference
	2:1 vs 1:2	0.11	−1.06 to 1.33	No clear subgroup difference
	1:1 vs 2:1	0.43	−0.42 to 1.28	No clear subgroup difference
Training frequency	3 sessions/week vs 2 sessions/week	0.40	−0.55 to 1.33	No clear subgroup difference

Stratification by training frequency. When studies were stratified by weekly training frequency, three sessions per week showed a positive pooled effect (g, 1.20, 95% CrI: 0.83–1.62), whereas the point estimate for two sessions per week was positive but its credible interval crossed zero (g, 0.80, 95% CrI: −0.03–1.67).

Stratification by work–recovery ratio. When studies were stratified by work–recovery ratio, the 1:1 protocol yielded the largest point estimate (g, 1.34, 95% CrI: 0.87–1.85), followed by the 2:1 protocol (g, 0.91, 95% CrI: 0.26–1.61). The 1:2 protocol showed a smaller and less stable effect (g, 0.81, 95% CrI: −0.16–1.76). These findings should be considered exploratory and should not be interpreted as evidence that a 1:1 structure is definitively superior.

Stratification by CRF outcome type. When studies were stratified by type of CRF outcome, the pooled effect size was 1.29 (95% CrI: 0.70–1.90) for VO_2_max, 1.18 (95% CrI: 0.64–1.76) for VO_2_peak, and 0.73 (95% CrI: −0.13–1.58) for the Yo-Yo test. These findings indicate that the VO_2_max and VO_2_peak subgroups showed larger point estimates and credible intervals that did not cross zero, whereas the subgroup using the field-based Yo-Yo test showed a smaller and less stable effect.

### 3.7 Sensitivity analyses

**Figure 5:**
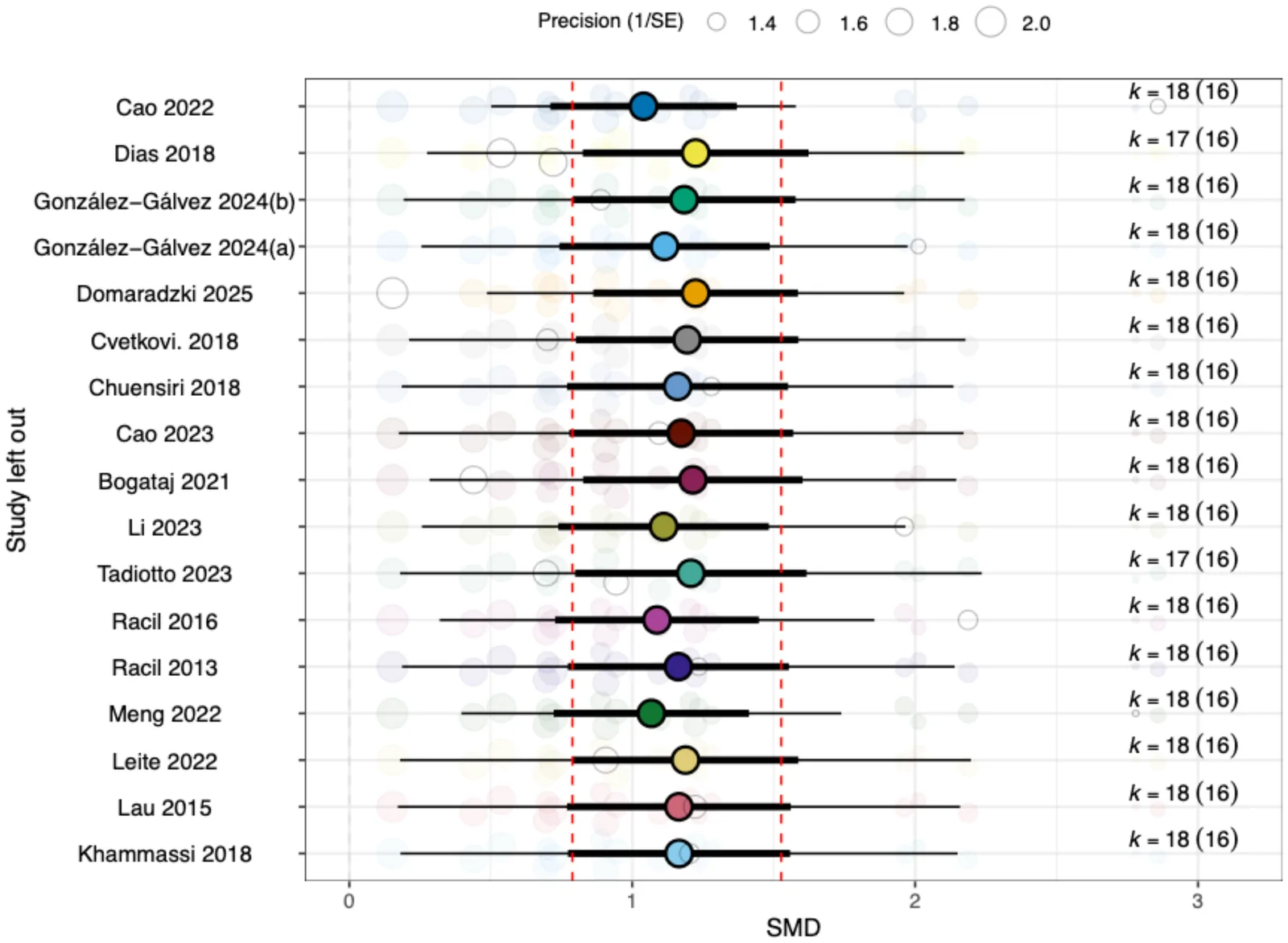
Sensitivity analyses result.

Sensitivity analyses using the leave-one-out approach showed that the direction of the pooled effect remained consistent after sequentially excluding each individual study, indicating that no single study drove the overall estimate (**Figure 5**). Prior-sensitivity analyses yielded similar pooled effects across alternative weakly informative priors (**Supplementary Table S6**). The primary model estimated a pooled effect of Hedges’ g, 1.13 (95% CrI: 0.80 to 1.50), and alternative prior specifications produced estimates ranging from 1.13 to 1.16. The corresponding 95% CrIs largely overlapped, indicating that the main conclusion was not materially affected by prior choice. All monitored parameters showed satisfactory convergence, with Rhat values close to 1.00 and adequate bulk-ESS and tail-ESS for the pooled effect and heterogeneity parameters.

### 3.8 Publication bias

**Figure 6:**
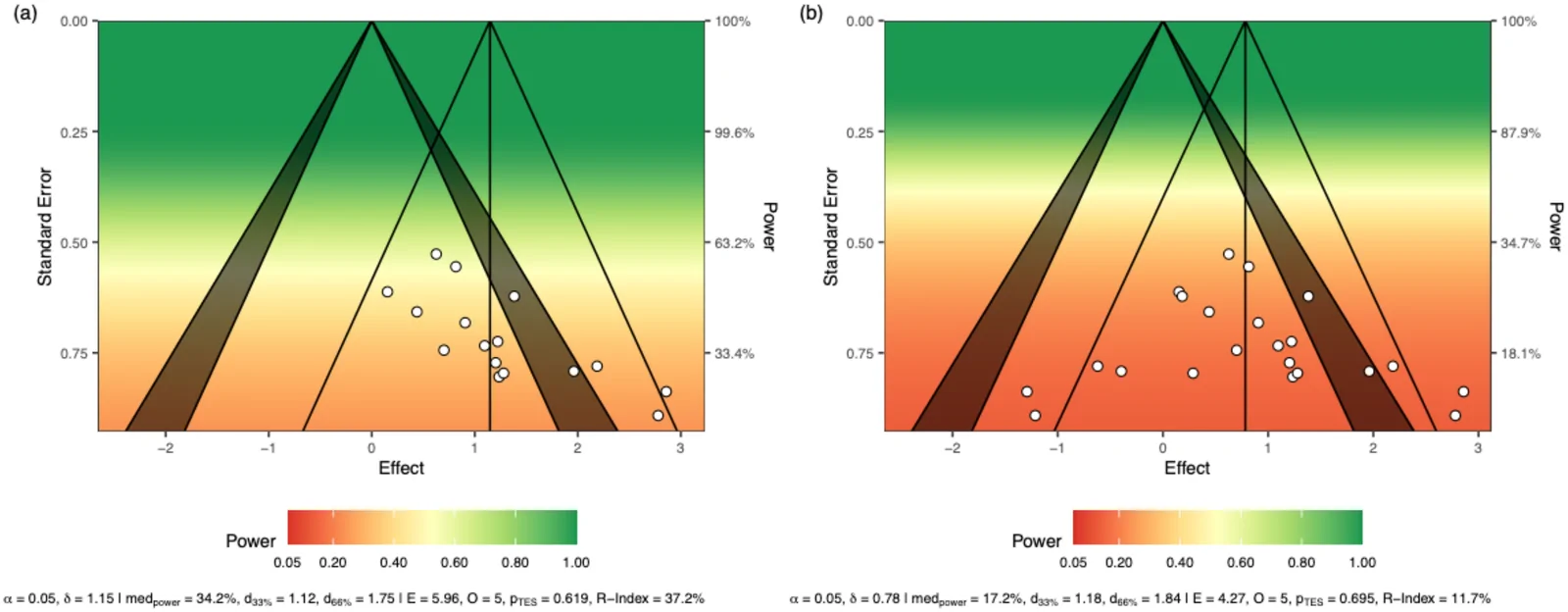
Funnel plot. **(a)**, Funnel plot of original results; **(b)** Funnel plot of trim-and-filled.

A funnel plot was generated for the CRF across the 16 studies. The effect sizes (Hedges’ g, 1.13) showed some asymmetry, prompting further investigation. Egger’s linear regression test was significant (intercept, -2.205, 95% CI: -4.71 to 0.29, p, 0.01), indicating the presence of publication bias. The trim-and-fill method estimated 6 missing studies on the left side of the funnel. After imputing these studies, the adjusted pooled effect remained positive and statistically significant, although the magnitude was attenuated compared with the unadjusted estimate (adjusted Hedges’ g, 0.78, 95% CI: 0.48–1.08, p < 0.001). The adjusted analysis showed moderate heterogeneity (I², 45.45%; Q (Bürkner, 2017), 38.49, p, 0.011) (**Figure 6**). After imputing these studies, the adjusted pooled effect remained positive and statistically significant, although the magnitude was attenuated compared with the unadjusted estimate (adjusted Hedges’ g, 0.78, 95% CI: 0.48–1.08, p < 0.001). The adjusted analysis showed moderate heterogeneity (I², 45.45%; Q (Bürkner, 2017), 38.49, p, 0.011). These findings suggest that the direction of the HIIT effect on CRF was robust, but the unadjusted pooled effect may have overestimated the magnitude of benefit.

## 4 Discussion

This study systematically reviewed and quantitatively synthesized the effects of HIIT on CRF in adolescents with overweight or obesity. The main analysis showed that HIIT was associated with a positive average effect on CRF in this population. This finding suggests that HIIT may improve CRF among adolescents with overweight or obesity compared with control conditions. Further exploratory subgroup analyses indicated that the effects of HIIT varied across training parameters. Exploratory dose–response analysis suggested that the association between weekly HIIT dose and CRF improvement was not simply linear, but may follow an inverted U-shaped curve, with the predicted peak at approximately 360 MET-min/week. Overall, this study not only provides further support for the beneficial effects of HIIT on CRF in adolescents with overweight or obesity, but also suggests that its intervention benefits may involve a possible moderate-dose window rather than a simple “more is better” pattern. This dose–response finding should be interpreted as hypothesis-generating rather than confirmatory.

The present findings are generally consistent with previous reviews. An earlier systematic review and meta-analysis by Costigan et al. reported that HIIT improved health-related fitness in adolescents, with particularly pronounced benefits for CRF (Costigan et al., 2015). Eddolls et al. further suggested that running-based, high-intensity HIIT performed two to three times per week for at least seven weeks was more likely to produce beneficial effects in children and adolescents (Eddolls et al., 2017). Martin-Smith et al., in a systematic review and meta-analysis involving healthy adolescents and adolescents with overweight or obesity, showed that HIIT significantly improved CRF (Martin-Smith et al., 2020). More recently, Deng and Wang focused on children and adolescents with overweight or obesity and further indicated that HIIT improved CRF in this population while beginning to address the issue of optimal dose (Deng and Wang, 2024). Compared with these studies, the present review focused specifically on adolescents with overweight or obesity, a high-risk population, and restricted the outcome to CRF; therefore, the research question was more focused and a larger intervention effect may have been more readily observed.

Importantly, most previous reviews have primarily addressed whether HIIT improves CRF or how HIIT compares with moderate-intensity continuous training or usual-care conditions. In contrast, fewer reviews have attempted to model the nonlinear dose–response association between HIIT dose and CRF improvement, particularly in adolescents with overweight or obesity. To the best of our knowledge, evidence specifically examining nonlinear weekly-dose patterns in this population remains limited. The present review therefore extends prior work by moving beyond the overall effectiveness question and by examining whether the magnitude of CRF improvement varies across weekly HIIT dose. This helps clarify not only whether HIIT may be beneficial, but also how prescription-related parameters may shape the expected response. Methodologically, this study also contributes to the literature by applying Bayesian multilevel modelling and spline-based dose–response modelling. Compared with conventional pairwise meta-analyses that mainly estimate an average intervention effect, this approach allowed us to account for dependent effect sizes from multi-arm trials, incorporate uncertainty in the pooled effect and heterogeneity parameters, and flexibly explore potential nonlinear dose–response patterns.

The large effect observed in this study may be related to the baseline characteristics of the study population. Adolescents with overweight or obesity often have relatively low baseline CRF, and CRF level and its improvement during youth are closely linked to future health outcomes. A systematic review and meta-analysis by García-Hermoso et al. showed that higher CRF during youth and improvements in CRF over time were associated with lower future risks of obesity and cardiometabolic risk (García-Hermoso et al., 2020). Johansson et al. also found in a clinical sample of children and adolescents with obesity that lower CRF was associated with adverse cardiometabolic characteristics, including higher high-sensitivity C-reactive protein (hs-CRP), and that this association persisted after adjustment for degree of obesity (Johansson et al., 2023). Because hs-CRP is a marker of low-grade systemic inflammation, this finding suggests that low CRF in adolescents with obesity may reflect not only reduced physical performance but also a less favorable inflammatory and cardiometabolic profile. Thus, in adolescents with overweight or obesity, improving CRF not only indicates enhanced exercise performance, but may also have potential clinical implications for inflammation and cardiometabolic risk.

From a physiological perspective, HIIT can induce high levels of cardiovascular and metabolic load within a relatively short period. Repeated high-intensity stimuli may improve VO_2_max or VO_2_peak by increasing maximal cardiac output, enhancing stroke volume, promoting skeletal-muscle capillarization, increasing mitochondrial oxidative capacity, and improving peripheral oxygen extraction efficiency. In adolescents with overweight or obesity, HIIT may also indirectly promote CRF improvements by improving insulin sensitivity, reducing inflammatory burden, and enhancing exercise self-efficacy (Cao et al., 2021; Duncombe et al., 2022). Although the primary outcome of this study was CRF, future studies should also examine body composition, inflammation, blood pressure, blood lipids, insulin resistance, and mental health to clarify the multisystem implications of CRF improvement.

Exploratory subgroup results suggested that HIIT performed three times per week was associated with a larger and more stable point estimate than HIIT performed twice per week. This trend is broadly consistent with previous reviews. Eddolls et al. suggested that two to three HIIT sessions per week is a common and potentially effective arrangement in children and adolescents (Eddolls et al., 2017). Deng et al. in their meta-analysis of children and adolescents with overweight or obesity, also reported that HIIT lasting more than 10 weeks, performed three times per week, with two to eight sets per session and a work–recovery ratio of approximately 1:1, was more likely to yield substantial CRF improvements (Deng and Wang, 2024). Therefore, three sessions per week may be considered a provisional practical reference rather than an established prescription standard, while still requiring adjustment according to individual obesity status, previous exercise experience, and safety monitoring.

With respect to the work–recovery structure, this study found that a 1:1 work–recovery ratio had larger point estimate, whereas 1:2 protocols showed less stable effects. A possible explanation is that a 1:1 structure maintains relatively high training density while allowing adequate recovery, thereby enabling participants to repeatedly reach the target intensity and obtain sufficient cardiopulmonary stimulation. Insufficient recovery may impair the quality of subsequent intervals, whereas excessively long recovery may dilute the training stimulus per unit time. Therefore, for adolescents with overweight or obesity whose primary goal is CRF improvement, recovery must be sufficient to maintain movement and intensity quality, but not so long that it weakens the training stimulus. However, because these findings were derived from a small body of evidence and exploratory subgroup analyses, they should not be interpreted as demonstrating the definitive superiority of a 1:1 work–recovery structure.

When CRF outcomes were stratified by measurement type, positive HIIT effects were more stable when CRF was assessed using VO_2_max or VO_2_peak, whereas effects were smaller and less stable when the Yo-Yo test was used. This suggests that CRF measurement method may be an important source of heterogeneity. VO_2_max/VO_2_peak are more direct and standardized physiological indicators, whereas field tests are more susceptible not only to aerobic capacity but also to motivation, running economy, skill familiarity, and testing organization. In adolescents with higher body weight, weight-bearing demands may additionally affect performance in field-running tests. Therefore, future studies and evidence syntheses should pay greater attention to consistency in outcome measurement, or at least treat measurement method as an important methodological factor when interpreting heterogeneous findings.

The moderate-to-substantial heterogeneity observed in the main analysis has important implications for interpretation. Although we explored several study-level moderators, none fully explained the between-study variance. This suggests that the pooled effect reflects an average across trials that differed in participant characteristics, baseline CRF, pubertal stage, obesity severity, intervention modality, supervision, adherence, comparator conditions, and CRF assessment methods. Some of these factors were incompletely reported or could only be coded at the study level, which limited the ability of moderator analyses to identify the sources of variability. Consequently, the pooled effect should not be interpreted as the expected response for every adolescent with overweight or obesity. Similarly, the estimated dose–response peak should not be viewed as a universal prescription threshold. Rather, the apparent peak around 360 MET-min/week represents a model-based average trend across heterogeneous studies and should be regarded as hypothesis-generating. Future trials with standardized reporting of HIIT prescription, adherence, supervision, and participant characteristics are needed to determine whether the dose–response relationship varies by sex, pubertal stage, baseline fitness, obesity severity, or intervention setting.

One exploratory finding of this study is the inverted U-shaped nonlinear relationship between HIIT dose and CRF improvement. This result suggests that, within the dose range covered by the included studies, a moderate-to-high weekly dose of HIIT may be most favorable for obtaining CRF benefits, whereas further increases in total training volume may not produce linear gains and may instead lead to diminishing marginal returns or slight attenuation of effects. Similarly, Deng et al. attempted to examine the optimal dose of HIIT in children and adolescents with overweight or obesity, emphasizing that “effective” and “optimal” are not equivalent in this field (Deng and Wang, 2024). The present study further provides a more intuitive estimate of the peak dose, but this estimate remains exploratory, model-dependent, and constrained by the dose range and reporting quality of the included trials. A plausible explanation is that low doses may provide insufficient stimulus to induce adaptation, whereas excessive doses may lead to accumulated fatigue, inadequate recovery, reduced completion quality, and lower adherence.

This study has several implications for public health practice. First, as a time-efficient form of exercise, HIIT is suitable for integration into after-school training, school physical education, or weight-management interventions. Second, the present findings and prior evidence suggest that, in practice, protocols involving approximately three sessions per week, a work–recovery ratio close to 1:1, and a moderate weekly dose may be considered as provisional starting points, with progressive adjustment according to each individual’s exercise background. These parameters should not be regarded as established prescription standards, because the supporting evidence comes mainly from exploratory subgroup analyses and indirect comparisons across a limited number of trials. Implementation should include safety screening, warm-up and cool-down, exercise-technique guidance, heart-rate or RPE monitoring, adverse-event recording, and strategies to support long-term adherence. Third, the dose–response findings highlight the importance of optimizing total weekly dose. For adolescents with overweight or obesity, prescription should emphasize safety, perceived tolerance, movement quality, and long-term sustainability, rather than merely pursuing higher intensity or longer duration.

This study also has several limitations. First, the overall sample size of the included studies was still relatively small, and the number of studies in some subgroups was limited, resulting in wide credible intervals and limited stability of some subgroup findings. Second, studies differed substantially in participant age, degree of obesity, training modality, recovery arrangement, and outcome measurement tools, which may have introduced methodological heterogeneity. Third, the conversion of training dose into MET-min/week relied on several assumptions, particularly regarding the MET assignment for work and recovery bouts, which may have introduced measurement error. Because the available information did not always allow precise reconstruction of individual-level exercise dose, the estimated dose–response peak may shift under alternative MET-coding assumptions. Fourth, multi-arm studies sharing a common control group may have generated correlated effect sizes, requiring appropriate statistical handling. Fifth, most existing studies used short- to medium-term interventions and lacked long-term follow-up; therefore, it remains unclear whether improvements in CRF are maintained over time and whether they translate into reductions in cardiometabolic event risk. Finally, Cochrane CENTRAL, trial registries, and grey literature sources were not searched. Therefore, unpublished, ongoing, or difficult-to-locate randomized controlled trials may have been missed, which could increase the possibility of publication bias or availability bias.

## 5 Conclusions

Current evidence suggests that HIIT may be an effective exercise strategy for improving CRF in adolescents with overweight or obesity. However, the magnitude of benefit should be interpreted cautiously because the included trials varied in participant characteristics, HIIT protocols, comparator conditions, and CRF assessment methods. Exploratory dose–response findings indicated a possible nonlinear association between weekly HIIT dose and CRF improvement, suggesting that a moderate weekly dose may be more favorable than simply increasing total training volume. This estimate should be regarded as hypothesis-generating rather than as a definitive optimal threshold, given the limited number of trials, residual heterogeneity, model dependence, and assumptions involved in MET-min/week coding. Future high-quality RCTs should use standardized exercise-reporting frameworks, such as FITT-VP or CERT, include larger samples and longer follow-up, and transparently report HIIT prescription, adherence, supervision, and adverse events to examine whether dose–response patterns vary by sex, pubertal stage, obesity severity, and baseline CRF level.

## Data Availability

The original contributions presented in the study are included in the article/**Supplementary Material**. Further inquiries can be directed to the corresponding author.
