# Correction: Effects of high-intensity interval training on cardiorespiratory fitness in adolescents with overweight or obesity: a systematic review and dose–response meta-analysis

**DOI:** 10.3389/fphys.2026.1996335

**Published:** 2026-10-07

**Authors:** Jinchen Pan, Xusong Dong, Ruiqi Cheng, Jianmei Cui, Weian Lin, Lei Zheng

**Affiliations:** 1 China Basketball College, Beijing Sport University, Beijing, China; 2 Strength And Conditioning Training College, Beijing Sports University, Beijing, China; 3 School of Athletic Performance, Shanghai University of Sport, Shanghai, China; 4 College of Physical Education, North University of China, Taiyuan, China; 5 The Key Laboratory of Adolescent Health Assessment and Exercise Intervention of the Ministry of Education, East China Normal University, Shanghai, China

**Keywords:** adolescents, cardiorespiratory fitness, dose–response meta-analysis, high-intensity interval training, obesity, overweight

The **Author contributions** statement was erroneously given as:

“JP: Conceptualization, Data curation, Formal analysis, Methodology, Software, Validation, Visualization, Writing – original draft. XD: Data curation, Writing – original draft. RC: Data curation, Methodology, Writing – original draft. JC: Methodology, Writing – original draft. LW: Methodology, Writing – original draft. LZ: Supervision, Writing – review & editing, Project administration.”

The correct **Author contributions** statement is:

“JP: Conceptualization, Methodology, Investigation, Data curation, Formal analysis, Writing – original draft. XD: Conceptualization, Methodology, Investigation, Data curation, Writing – original draft, Writing – review & editing. RC: Investigation, Writing – review & editing. JC: Investigation, Writing – review & editing. WL: Investigation, Writing – review & editing. LZ: Methodology, Investigation, Data curation, Supervision, Writing – review & editing.”

The equal contribution statement for “Jinchen Pan and Xusong Dong” was missing from the published article. The correct statement is:

“These authors have contributed equally to this work and share first authorship”

The original version of this article has been updated.

