# Supplementary material for: Effects of high-intensity interval training on cardiorespiratory fitness in adolescents with overweight or obesity: a systematic review and dose–response meta-analysis
Source: Front Physiol. 2026 Aug 18;17:1885059. doi: 10.3389/fphys.2026.1885059 (PMC13529989; doi:10.3389/fphys.2026.1885059)
Supplement: Supplementary file 1 [file Table1.docx]

| Database | Search strategy | Results |
| --- | --- | --- |
| Web of Science | TS=(  ("high-intensity interval training" OR "high intensity interval training" OR HIIT OR "high-intensity intermittent" OR "high intensity intermittent" OR "sprint interval training" OR SIT OR "aerobic interval training" OR "interval exercise" OR "interval training")  AND  ("cardiorespiratory fitness" OR CRF OR "maximal oxygen uptake" OR "peak oxygen uptake" OR VO2max OR "VO2 max" OR VO2peak OR "VO2 peak" OR "aerobic capacity" OR "cardiovascular fitness")  AND  (adolescen* OR youth* OR teen* OR pubert* OR "high school student*" OR "secondary school student*")  AND  (obes* OR overweight OR adiposity OR "body weight")  ) | 213 |
| PubMed | ( "High-Intensity Interval Training"[Mesh] OR "high-intensity interval training"[Title/Abstract] OR "high intensity interval training"[Title/Abstract] OR HIIT[Title/Abstract] OR "high-intensity intermittent"[Title/Abstract] OR "high intensity intermittent"[Title/Abstract] OR "sprint interval training"[Title/Abstract] OR SIT[Title/Abstract] OR "aerobic interval training"[Title/Abstract] OR "interval exercise"[Title/Abstract] OR "interval training"[Title/Abstract] ) AND ( "Cardiorespiratory Fitness"[Mesh] OR "Physical Fitness"[Mesh] OR "Oxygen Consumption"[Mesh] OR "cardiorespiratory fitness"[Title/Abstract] OR CRF[Title/Abstract] OR "maximal oxygen uptake"[Title/Abstract] OR "peak oxygen uptake"[Title/Abstract] OR VO2max[Title/Abstract] OR "VO2 max"[Title/Abstract] OR VO2peak[Title/Abstract] OR "VO2 peak"[Title/Abstract] OR "aerobic capacity"[Title/Abstract] OR "cardiovascular fitness"[Title/Abstract] ) AND ( "Adolescent"[Mesh] OR adolescent[Title/Abstract] OR adolescents[Title/Abstract] OR youth[Title/Abstract] OR youths[Title/Abstract] OR teenager[Title/Abstract] OR teenagers[Title/Abstract] OR teen[Title/Abstract] OR teens[Title/Abstract] OR puberty[Title/Abstract] OR pubertal[Title/Abstract] OR "high school student"[Title/Abstract] OR "high school students"[Title/Abstract] OR "secondary school student"[Title/Abstract] OR "secondary school students"[Title/Abstract] ) AND ( "Obesity"[Mesh] OR "Overweight"[Mesh] OR "Adiposity"[Mesh] OR "Body Weight"[Mesh] OR obesity[Title/Abstract] OR obese[Title/Abstract] OR overweight[Title/Abstract] OR adiposity[Title/Abstract] OR "body weight"[Title/Abstract] ) | 295 |
| Scopus | TITLE-ABS-KEY ( ( "high-intensity interval training" OR "high intensity interval training" OR HIIT OR "high-intensity intermittent" OR "high intensity intermittent" OR "sprint interval training" OR SIT OR "aerobic interval training" OR "interval exercise" OR "interval training" ) AND ( "cardiorespiratory fitness" OR CRF OR "maximal oxygen uptake" OR "peak oxygen uptake" OR VO2max OR "VO2 max" OR VO2peak OR "VO2 peak" OR "aerobic capacity" OR "cardiovascular fitness" ) AND ( adolescen* OR youth* OR teen* OR pubert* OR "high school student*" OR "secondary school student*" ) AND ( obes* OR overweight OR adiposity OR "body weight" ) ) | 291 |
| Embase | (  'high intensity interval training'/exp  OR 'high-intensity interval training':ti,ab,kw  OR 'high intensity interval training':ti,ab,kw  OR HIIT:ti,ab,kw  OR 'high-intensity intermittent':ti,ab,kw  OR 'high intensity intermittent':ti,ab,kw  OR 'sprint interval training':ti,ab,kw  OR SIT:ti,ab,kw  OR 'aerobic interval training':ti,ab,kw  OR 'interval exercise':ti,ab,kw  OR 'interval training':ti,ab,kw  )  AND  (  'cardiorespiratory fitness'/exp  OR 'oxygen consumption'/exp  OR 'physical fitness'/exp  OR 'cardiorespiratory fitness':ti,ab,kw  OR CRF:ti,ab,kw  OR 'maximal oxygen uptake':ti,ab,kw  OR 'peak oxygen uptake':ti,ab,kw  OR VO2max:ti,ab,kw  OR 'VO2 max':ti,ab,kw  OR VO2peak:ti,ab,kw  OR 'VO2 peak':ti,ab,kw  OR 'aerobic capacity':ti,ab,kw  OR 'cardiovascular fitness':ti,ab,kw  )  AND  (  'adolescent'/exp  OR adolescen*:ti,ab,kw  OR youth*:ti,ab,kw  OR teen*:ti,ab,kw  OR pubert*:ti,ab,kw  OR 'high school student*':ti,ab,kw  OR 'secondary school student*':ti,ab,kw  )  AND  (  'obesity'/exp  OR 'overweight'/exp  OR 'body weight'/exp  OR 'adiposity'/exp  OR obes*:ti,ab,kw  OR overweight:ti,ab,kw  OR adiposity:ti,ab,kw  OR 'body weight':ti,ab,kw  ) | 137 |
| CNKI | SU=('高强度间歇训练' OR '高强度间歇运动' OR '高强度间歇性训练' OR '高强度间歇性运动' OR 'HIIT' OR '冲刺间歇训练' OR 'SIT' OR '有氧间歇训练' OR '间歇运动' OR '间歇训练')  AND  SU=('心肺适能' OR '心肺耐力' OR '心肺功能' OR '最大摄氧量' OR '峰值摄氧量' OR 'VO2max' OR 'VO2 max' OR 'VO2peak' OR 'VO2 peak' OR '有氧能力' OR '心血管适能')  AND  SU=('青少年' OR '少年' OR '青年' OR '中学生' OR '初中生' OR '高中生' OR '青春期')  AND  SU=('肥胖' OR '超重' OR '肥胖症' OR '体重' OR '身体脂肪' OR '体脂' OR '脂肪量') | 26 |
