# Supplementary material for: Effects of high-intensity interval training on cardiorespiratory fitness in adolescents with overweight or obesity: a systematic review and dose–response meta-analysis
Source: Front Physiol. 2026 Aug 18;17:1885059. doi: 10.3389/fphys.2026.1885059 (PMC13529989; doi:10.3389/fphys.2026.1885059)
Supplement: Supplementary file 3 [file Table3.docx]

Table S3. Hierarchical rules for coding weekly HIIT dose as MET-min/week

| **Priority** | **Available information in the original study** | **Coding rule** | **Application and assumptions** |
| --- | --- | --- | --- |
| 1 | Directly reported MET values, energy expenditure, or oxygen-cost estimates | Use the study-reported value | Applied separately to work and recovery bouts where available; no additional conversion was performed |
| 2 | Exercise modality with detailed external workload, such as running speed, cycling power, treadmill grade, or ergometer workload | Match the reported modality and workload to the closest 2011 Compendium activity code | Used when objective workload was available but MET values were not directly reported |
| 3 | Physiological intensity targets, such as %HRmax, %HRR, %VO₂max, or %VO₂peak | Convert the reported physiological target into an intensity category and match it to the closest Compendium code for the exercise modality | Heart-rate- or oxygen-uptake-based indicators were prioritized over subjective indicators |
| 4 | Rating of perceived exertion (RPE) or descriptive intensity targets, such as “near maximal,” “vigorous,” or “all-out” | Convert RPE or descriptive intensity into an intensity category, then assign the closest conservative MET value based on modality | Used only when direct MET, workload, or physiological intensity indicators were unavailable |
| 5 | Recovery-bout description, such as passive rest, standing rest, walking, slow running, or low-intensity cycling | Assign recovery MET according to the recovery modality and intensity | Passive recovery and active recovery were coded separately; active recovery was not treated as zero dose |
| 6 | Progressive protocols with changes in interval number, work duration, recovery duration, or intensity across weeks | Calculate weekly dose for each reported phase and use the average weekly dose across the intervention period | Used to avoid over-representing either the initial or final training load |
| 7 | Insufficient intensity information beyond general HIIT description | Assign a conservative MET value based on the exercise modality and minimum reported intensity compatible with HIIT | These cases were flagged as assumption-dependent in the supplementary coding table |
