# Supplementary material for: Effects of high-intensity interval training on cardiorespiratory fitness in adolescents with overweight or obesity: a systematic review and dose–response meta-analysis
Source: Front Physiol. 2026 Aug 18;17:1885059. doi: 10.3389/fphys.2026.1885059 (PMC13529989; doi:10.3389/fphys.2026.1885059)
Supplement: Supplementary file 4 [file Table4.docx]

Table S4. Bayesian model diagnostics

| **Model** | **variable** | **mean** | **sd** | **q025** | **q975** | **rhat** | **ess_bulk** | **ess_tail** |
| --- | --- | --- | --- | --- | --- | --- | --- | --- |
| Primary | b_Intercept | 1.13 | 0.18 | 0.80 | 1.50 | 1.00 | 7873.10 | 8240.48 |
| Primary | sd_Study_ID__Intercept | 0.46 | 0.23 | 0.04 | 0.91 | 1.00 | 1458.16 | 2551.90 |
| Primary | sd_Study_ID:esid__Intercept | 0.28 | 0.20 | 0.01 | 0.74 | 1.00 | 1619.47 | 4416.25 |
| Wide_effect_prior | b_Intercept | 1.16 | 0.18 | 0.81 | 1.55 | 1.00 | 6133.01 | 7693.70 |
| Wide_effect_prior | sd_Study_ID__Intercept | 0.45 | 0.23 | 0.03 | 0.90 | 1.00 | 1541.01 | 2386.66 |
| Wide_effect_prior | sd_Study_ID:esid__Intercept | 0.30 | 0.21 | 0.01 | 0.77 | 1.00 | 1536.71 | 3920.06 |
| Wide_heterogeneity_prior | b_Intercept | 1.14 | 0.18 | 0.79 | 1.51 | 1.00 | 6495.27 | 8684.70 |
| Wide_heterogeneity_prior | sd_Study_ID__Intercept | 0.48 | 0.24 | 0.04 | 0.95 | 1.00 | 1460.48 | 2398.63 |
| Wide_heterogeneity_prior | sd_Study_ID:esid__Intercept | 0.31 | 0.22 | 0.01 | 0.78 | 1.00 | 1443.78 | 3941.26 |
| Halfnormal_heterogeneity_prior | b_Intercept | 1.13 | 0.17 | 0.80 | 1.49 | 1.00 | 7393.91 | 7697.33 |
| Halfnormal_heterogeneity_prior | sd_Study_ID__Intercept | 0.45 | 0.21 | 0.04 | 0.86 | 1.00 | 2017.33 | 3409.49 |
| Halfnormal_heterogeneity_prior | sd_Study_ID:esid__Intercept | 0.30 | 0.20 | 0.01 | 0.73 | 1.00 | 1976.49 | 4992.54 |
