# Supplementary material for: Effects of high-intensity interval training on cardiorespiratory fitness in adolescents with overweight or obesity: a systematic review and dose–response meta-analysis
Source: Front Physiol. 2026 Aug 18;17:1885059. doi: 10.3389/fphys.2026.1885059 (PMC13529989; doi:10.3389/fphys.2026.1885059)
Supplement: Supplementary file 5 [file Table5.docx]

Table S5. Comparison of alternative nonlinear dose–response models using approximate leave-one-out cross-validation

| **Model** | **Nonlinear specification** | **elpd_diff** | **SE of elpd_diff** | **elpd_loo** | **SE of elpd_loo** | **p_loo** | **SE of p_loo** | **LOOIC** | **SE of LOOIC** |
| --- | --- | --- | --- | --- | --- | --- | --- | --- | --- |
| model_rcs4 | Restricted cubic spline, 4 knots | 0.0 | 0.0 | -20.1 | 3.1 | 12.8 | 1.9 | 40.2 | 6.2 |
| model_smooth_k4 | Penalized spline smooth, k = 4 | -0.1 | 0.7 | -20.2 | 3.4 | 12.5 | 2.0 | 40.3 | 6.8 |
| model_rcs3 | Restricted cubic spline, 3 knots | -0.1 | 0.6 | -20.2 | 3.3 | 12.9 | 2.0 | 40.4 | 6.5 |
| model_smooth_k3 | Penalized spline smooth, k = 3 | -0.2 | 0.8 | -20.2 | 3.4 | 12.6 | 2.1 | 40.5 | 6.8 |
| model_poly2 | Quadratic polynomial | -0.6 | 0.9 | -20.7 | 3.5 | 12.9 | 2.2 | 41.4 | 7.1 |
| model_log | Log-dose model | -0.7 | 1.2 | -20.8 | 3.6 | 13.1 | 2.2 | 41.6 | 7.1 |
| model_ns3 | Natural spline, df/k = 3 | -0.9 | 0.8 | -21.0 | 3.3 | 13.4 | 2.1 | 42.0 | 6.7 |
| model_bs3 | B-spline, df/k = 3 | -1.1 | 0.9 | -21.2 | 3.3 | 13.6 | 2.2 | 42.4 | 6.7 |
| model_bs4 | B-spline, df/k = 4 | -1.5 | 1.5 | -21.6 | 3.7 | 13.5 | 2.0 | 43.3 | 7.5 |
| model_poly3 | Cubic polynomial | -1.6 | 0.8 | -21.7 | 3.2 | 14.0 | 2.1 | 43.5 | 6.5 |
| model_ns4 | Natural spline, df/k = 4 | -2.1 | 1.5 | -22.2 | 3.8 | 14.2 | 2.1 | 44.3 | 7.5 |

Models were ranked by expected log predictive density estimated using approximate leave-one-out cross-validation. Higher elpd_loo and lower LOOIC indicate better expected out-of-sample predictive performance. The four-knot restricted cubic spline model showed the best predictive performance, but its advantage over the k = 4 penalized spline, three-knot restricted cubic spline, and k = 3 penalized spline models was negligible relative to the standard errors of the differences. Therefore, the dose–response pattern should be interpreted as exploratory and model-dependent rather than as evidence of a definitive optimal threshold.
