# Supplementary material for: Effects of high-intensity interval training on cardiorespiratory fitness in adolescents with overweight or obesity: a systematic review and dose–response meta-analysis
Source: Front Physiol. 2026 Aug 18;17:1885059. doi: 10.3389/fphys.2026.1885059 (PMC13529989; doi:10.3389/fphys.2026.1885059)
Supplement: Supplementary file 6 [file Table6.docx]

Table S6. Prior-sensitivity analysis of the primary Bayesian meta-analysis

| **Model** | **Prior for pooled effect** | **Prior for heterogeneity** | **Hedges’ g** | **CrI_lower** | **CrI_upper** | **Tau_total** | **Tau_lower** | **Tau_upper** |
| --- | --- | --- | --- | --- | --- | --- | --- | --- |
| Primary | Normal(0,1) | Half-Cauchy(0,0.5) | 1.13 | 0.80 | 1.50 | 0.60 | 0.32 | 0.97 |
| Wide effect prior | Normal(0,2) | Half-Cauchy(0,0.5) | 1.16 | 0.81 | 1.55 | 0.60 | 0.33 | 0.96 |
| Wide heterogeneity prior | Normal(0,1) | Half-Cauchy(0,1) | 1.13 | 0.79 | 1.51 | 0.63 | 0.35 | 1.03 |
| Half-normal heterogeneity prior | Normal(0,1) | Half-Normal(0,0.5) | 1.13 | 0.80 | 1.49 | 0.59 | 0.32 | 0.93 |
